# Tacrolimus to belatacept conversion in proteinuric kidney transplant recipients

**DOI:** 10.3389/fimmu.2024.1491514

**Published:** 2024-12-23

**Authors:** Orhan Efe, Ayman Al Jurdi, Morgan Mabey Eiting, Christine Rogers Marks, Mariesa Ann Cote, David Wojciechowski, Kassem Safa, Hannah Gilligan, Jamil Azzi, Nitender Goyal, Marc Raynaud, Alexandre Loupy, Astrid Weins, Leonardo V. Riella

**Affiliations:** ^1^ Division of Nephrology, Department of Medicine, Massachusetts General Hospital, Boston, MA, United States; ^2^ Solid Organ Transplant Pharmacy, Massachusetts General Hospital, Boston, MA, United States; ^3^ Kidney Transplantation Program, UT Southwestern Medical Center, Dallas, TX, United States; ^4^ Transplantation Research Center, Renal Division, Brigham and Women’s Hospital, Boston, MA, United States; ^5^ Division of Nephrology, Tufts Medical Center, Boston, MA, United States; ^6^ Paris Translational Research Center for Organ Transplantation, INSERM, Paris Cardiovascular Research Center, Université de Paris, Paris, France; ^7^ Department of Pathology, Brigham and Women’s Hospital, Boston, MA, United States; ^8^ Center for Transplantation Sciences, Surgery Department, Massachusetts General Hospital, Harvard Medical School, Boston, MA, United States

**Keywords:** belatacept conversion, kidney transplantation, proteinuria, graft function, proteinuria reduction

## Abstract

**Background:**

Proteinuria is associated with worse allograft outcomes in kidney transplant recipients (KTRs) and treatment strategies are limited. We examined the outcomes of calcineurin inhibitor (CNI) to belatacept conversion in proteinuric KTRs.

**Methods:**

In a pilot phase II single-arm multicenter prospective trial, we recruited adult KTRs >6 months post-kidney transplantation with an estimated glomerular filtration rate (eGFR) ≥30 ml/min/1.73m^2^ and proteinuria >1 g/day. Patients were converted from CNI to belatacept. The primary outcome was a 25% reduction in proteinuria at 12 months.

**Results:**

A total of 15 KTRs were recruited who had pre-conversion median (interquartile range) proteinuria of 1.8 (IQR 1.4 – 3.5) g/g and estimated glomerular filtration rate (eGFR) of 48 (IQR 32 – 52.5) ml/min/1.73m^2^. At 12 months post-conversion, median proteinuria was 1.4 (IQR 0.4 – 2.2) g/g (*P* = 0.068) and eGFR was maintained at 43 (34 – 54.5) ml/min/1.73m^2^. The primary outcome of at least a 25% reduction in proteinuria occurred in 53% (8/15) at 12 months. Abbreviated IBOX scores predicting 7-year graft survival were also stable at 1-year post-conversion compared to baseline. At extended follow-up at 5 years, both proteinuria and eGFR remained stable at 0.69 (0.24 – 2.15) g/g and 39 (31 – 57) ml/min/1.73m^2^, respectively.

**Conclusions:**

CNI to belatacept conversion was associated with preserved allograft function in KTRs with significant proteinuria. These findings need to be confirmed in a larger randomized clinical trial.

**Clinical trial registration:**

https://clinicaltrials.gov/, **identifier NCT0232740.**

## 1 Introduction

Proteinuria is associated with reduced allograft and patient survival in kidney transplant recipients (KTRs) (1, 2). In KTRs on calcineurin inhibitors, optimization of medications that block the renin-angiotensin-aldosterone system (RAAS) is frequently limited by adverse effects such as hyperkalemia (3, 4). Furthermore, no randomized controlled trials have investigated the anti-proteinuric effects of SGLT-2 inhibitors in KTRs. Therefore, additional strategies are needed to reduce proteinuria and prolong allograft survival in KTRs with proteinuria.

In patients with podocytopathies and glomerulonephritis, calcineurin inhibitors (CNIs) reduce proteinuria through immune and non-immune effects such as vasoconstriction and podocyte stabilizing effects (5). On the other hand, they can also cause proteinuria through a variety of mechanisms, including tubular injury, thrombotic microangiopathy, and glomerulosclerosis (6–9). - CNIs can also impair endothelial function through oxidative stress and vasoconstriction, further contributing to glomerular injury and proteinuria. In contrast, belatacept does not share these vasoactive properties, potentially supporting healthier endothelium and reduced proteinuria. Some preclinical studies postulated the anti-proteinuric effects of costimulation blockade (10, 11). In a retrospective cohort of proteinuric KTRs, belatacept conversion from CNIs or mammalian target of rapamycin (mTOR) inhibitors was associated with reduced proteinuria at 12 months post-conversion (7). However, this has not been investigated prospectively as KTRs with high-grade proteinuria were excluded from clinical trials of belatacept (12, 13). In this study, we conducted a phase II interventional pilot trial to evaluate the effect of CNI to belatacept conversion in KTRs with high-grade proteinuria.

## 2 Methods

### 2.1 Study design and participants

The study was an open-label, single-arm, interventional phase II trial, which enrolled proteinuric KTRs and converted their immunosuppression from CNI to belatacept-based maintenance therapy. Study endpoints were evaluated at 12 months post-conversion. The patients were enrolled at two centers: Brigham and Women’s Hospital (BWH) and Massachusetts General Hospital (MGH), Boston, Massachusetts, between 2016-2019. Inclusion criteria were KTRs who are: 1) ≥18-year-old and >6 months post-transplantation, 2) Epstein-Barr virus (EBV) IgG positive, 3) on CNI-based immunosuppression with an antiproliferative agent with or without glucocorticoids, 4) have proteinuria of ≥ 1g/g on spot urine protein-to-creatinine ratio (UPCR), and 5) an estimated glomerular filtration rate (eGFR) ≥30 mL/min/1.73 m^2^. Exclusion criteria included age <18 years, eGFR <30 ml/min/1.73 m^2^, active acute cellular rejection (ACR; higher than borderline) or ACR within the last 6 months, active acute antibody-mediated rejection, recurrent primary focal segmental glomerulosclerosis (FSGS), EBV IgG negative KTRs, current mTOR inhibitor use, and patients only on CNI (cyclosporine or tacrolimus) and glucocorticoids. All patients had an available kidney biopsy within a year of enrollment. The study was registered in the ClinicalTrials.gov database (NCT02327403). Recruitment was interrupted early due to the COVID-19 pandemic in 2020.

Patients were seen in clinical visits at 0, 1, 3, 6, 9, and 12 months. Serum creatinine and UPCR were collected during the clinical visits. Laboratory data, including lipid panel and random glucose measurements, were collected at baseline and 12 months. Data from KTRs who remained on belatacept beyond 12 months were collected in an observational manner by chart review.

### 2.2 Study endpoints

The primary endpoint was the efficacy of belatacept conversion in reducing proteinuria by at least 25% at 12 months. Secondary endpoints included change in eGFR from baseline to 12 months, adverse events, acute rejection episodes, death-censored graft survival, patient survival, and changes in blood pressure, BMI, glucose, and lipid levels at 12 months.

Pre- and post-conversion abbreviated IBOX scores were calculated as an exploratory outcome based on pre- vs post-conversion eGFR and proteinuria values. The IBOX score is a risk prediction score for kidney allograft failure based on clinical characteristics, including eGFR, proteinuria, and allograft biopsy findings (14). The abbreviated IBOX score uses only functional parameters and has shown good prediction performances. Both full and abbreviated IBOX are currently approved by the European Medicines Agency (EMA) to serve as a surrogate endpoint for clinical trials and are under review by Food and Drug Administration (FDA) (15). The 2021 Chronic Kidney Disease Epidemiology Collaboration (CKD-EPI) calculator without race was used for eGFR calculations.

### 2.3 Procedure

After study enrollment, belatacept was administrated at 5 mg/kg IV on days 1, 15, 29, 43, and 57, followed by every 28 days thereafter. CNI was maintained at the same dose for 1 week from belatacept initiation, then tapered weekly by 25% and discontinued at the end of week 4. Adjustments of other medications, including ACEIs and ARBs, were managed at the discretion of the caring physician.

### 2.4 Statistics

Mean with standard deviation (SD) or median with interquartile ranges (IQR) were used to describe continuous variables depending on the normality of distribution. Frequencies and percentiles were used for categorical variables. For paired comparisons of continuous variables, paired t-test or Wilcoxon signed-rank test were used as appropriate. For unpaired comparisons of continuous variables, two-tailed unpaired t test or Mann-Whitney U-test were used depending on data distribution.

### 2.5 Study approval

This study was approved by the institutional review board at Mass General Brigham (protocol #: 2015P000154). All subjects in the prospective study signed written informed consent. The research activities were conducted in compliance with the Declaration of Helsinki.

## 3 Results

### 3.1 Study participants

A total of 15 patients were enrolled in the prospective interventional trial, 13 of 15 completed 12 months of follow-up. Eighty percent of the patients were negative for donor-specific antibodies (DSA) at baseline. The mean time from transplant to belatacept conversion was 78 ± 67 months. Pre-conversion, median eGFR was 48 (IQR 32 – 52.5) mL/min/1.73m^2^, and median proteinuria was 1.8 (IQR 1.5-3.5) g/g. Secondary FSGS and diabetic nephropathy were the most common causes of proteinuria identified at pre-enrollment kidney biopsy. Ten of 15 patients (67%) were on angiotensin-converting enzyme inhibitors (ACEI) or angiotensin receptor blockers (ARB) at baseline. The other patients were not able to use ACEI or ARB due to side effects such as hyperkalemia. Baseline characteristics are summarized in **Table 1**.

**Table 1:** Patients’ demographics and characteristics.

Patients’ Demographics and Characteristics (n=15)	Median (IQR) or N (%)
Age in years	61 (50 – 65)
Male	13 (87)
Ethnicity Black White Asian Hispanic	3 (20) 7 (47) 2 (13) 3 (20)
Living kidney transplant	4 (27)
BMI, kg/m^2^	29.6 (26.9 – 35.7)
Number of HLA mismatches (ABDR)	5 (2 – 5)
Pre-conversion DSA Negative Positive Unavailable	12 (80) 2 (13) 1 (7)
Pre-transplant Comorbidities Hypertension Diabetes Mellitus	11 (73) 6 (40)
Etiology of end-stage kidney disease Diabetic nephropathy IgA nephropathy Hypertensive nephropathy Primary FSGS Anti-GBM disease Other*	4 (26) 3 (20) 2 (13) 1 (7) 1 (7) 4 (26)
Biopsy findings related to proteinuria Secondary FSGS Diabetic nephropathy * De novo* or recurrent glomerulonephritis Transplant glomerulopathy Chronic rejection	9 (60) 6 (40) 5 (33) 2 (13) 1 (7)
Baseline immunosuppression CNI Mycophenolate or mycophenolic acid Azathioprine Prednisone	15 (100) 14 (93) 1 (7) 9 (60)
Time to Belatacept conversion (months)	55.5 (33.2 – 129.7)
Baseline creatinine, mg/dL	1.56 (1.31 – 2.02)
Baseline eGFR, ml/min/1.73m^2^	48 (32 – 52.5)
Baseline UPCR, g/g	1.8 (1.45 – 3.54)
ACEI or ARB use	10 (67)

*Other etiologies include prune belly syndrome, collapsing FSGS in the setting of obesity and hypertension, traumatic laceration in a patient with solitary kidney, and an unknown etiology; n=1 for each. ACEI, angiotensin converting enzyme inhibitor; ARB, angiotensin receptor blocker; BMI, body mass index; CNI, calcineurin inhibitor; DSA, donor-specific antibody; eGFR, estimated glomerular filtration rate; FSGS, focal segmental glomerulosclerosis; HLA, human leukocyte antigen; UPCR, urine protein to creatinine ratio.

### 3.2 Efficacy outcomes

**Figure 1:**
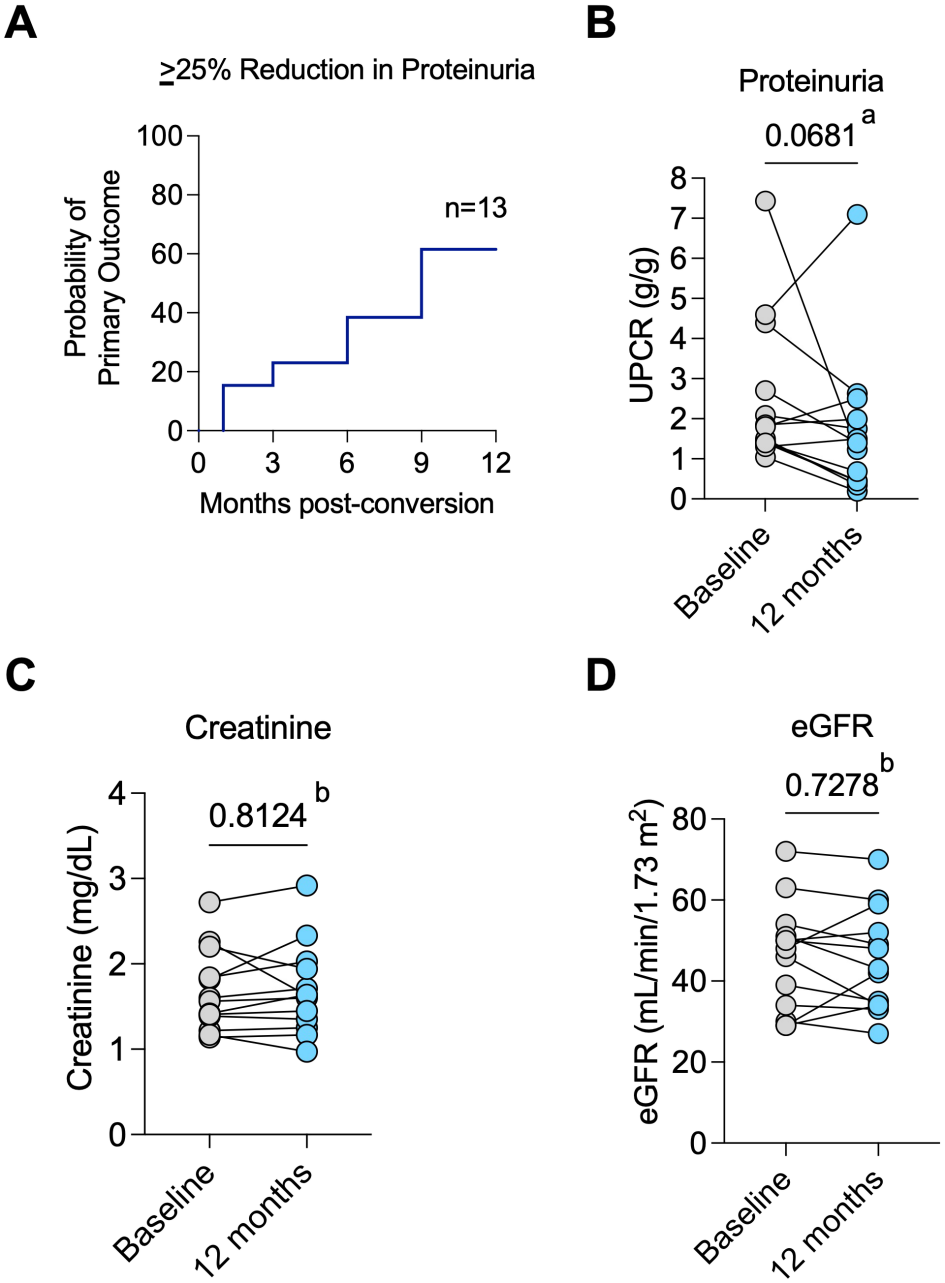
Primary outcome and allograft function. **(A)** Probability of achieving the primary outcome of ≥25% reduction in proteinuria over time; **(B)** Change in proteinuria from baseline to 12 months post-conversion for individual patients; **(C)** Changes in serum creatinine values and **(D)** estimated glomerular filtration rate (eGFR) from baseline to post-conversion 12 months for each patient. ^a^ Wilcoxon test. ^b^ Paired t test.

The primary outcome of at least a 25% reduction in proteinuria occurred in 8 of 15 patients (53%) at 12 months post-belatacept conversion (**Figure 1A**). Median proteinuria was 1.8 (IQR 1.4-3.5) g/g at baseline and 1.4 (0.4-2.5) g/g (*P* = 0.068) at 12 months (**Figure 1B**). Median percent reductions in proteinuria were 20% (IQR -75 and 33%) and 51% (IQR -76 and -17.5%) in diabetic vs non-diabetic patients, respectively (*P* = 0.3422).

**Figure 2:**
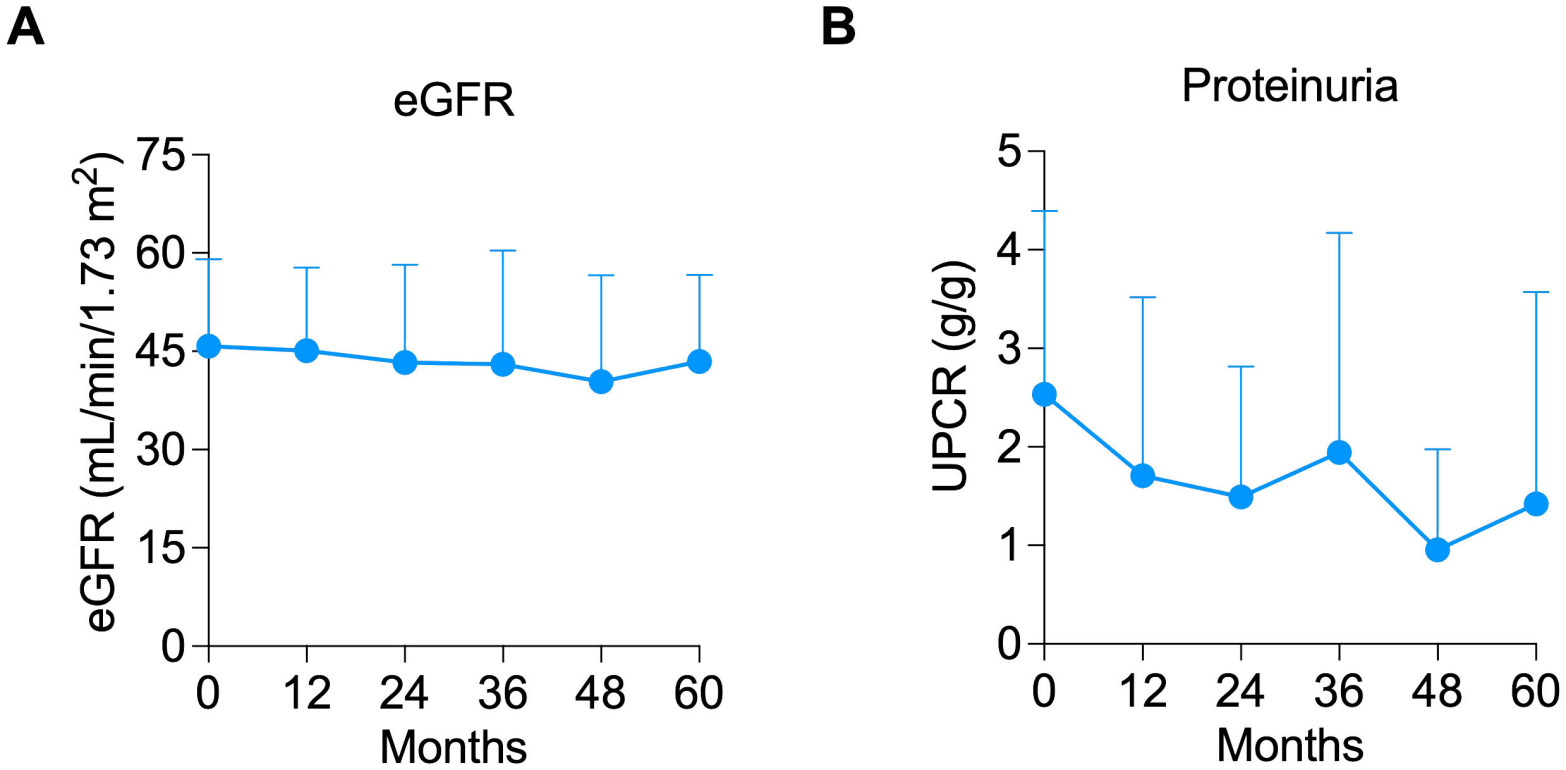
Long-term outcomes of allograft function and proteinuria. Line graph showing the maintenance of **(A)** estimated glomerular filtration rate (eGFR) and **(B)** proteinuria throughout the years.

Two patients were able to newly start ACEI/ARB post-belatacept conversion. However, it is important to note that both patients had experienced a reduction in proteinuria after belatacept conversion and *prior* to starting RAAS blockers. Another patient had the ACEI discontinued within the first month of the study but still had an 81% reduction in proteinuria at 12 months. Median creatinine (**Figure 1C**) and eGFR (**Figure 1D**) were similar at pre-conversion and 12 months post-conversion (eGFR of 48 (IQR 32 – 52.5) vs. 43 (34 – 54.5) mL/min/1.73m^2^, respectively, *P* = 0.728). Predicted 7-year graft survival was 74.2% (IQR 61.9 – 71.8) pre-conversion and remained stable at 73.5% (IQR 60.7 – 86.1) at 12 months post-conversion (*P* = 0.455, n=13, **Supplementary Figure S1A**). After 12 months, all 13 patients who completed 12 months of the study remained on belatacept until the last follow-up for a median of 5 (IQR 3- 5) years. Stable eGFR (**Figure 2A**) and proteinuria (**Figure 2B**) were maintained throughout the observation period.

Metabolic parameters are summarized in **Supplementary Table S1**. There was no significant change in blood pressure, total cholesterol, low-density lipoprotein, blood glucose, or triglyceride levels post-belatacept conversion. High-density lipoprotein (HDL) levels decreased from 47 (IQR 37 – 65) at baseline to 38 (IQR 31 – 49) mg/dL post-conversion (*P* = 0.036).

### 3.3 Safety outcomes

In the first year, none of the patients developed acute rejection (**Supplementary Figure S2A**), and death-censored graft survival (**Supplementary Figure S2B**) was 100%. One patient had worsening proteinuria post-belatacept conversion and discontinued belatacept at 3 months. One patient had a sudden cardiac arrest and died from an unidentified cause at 4 months (**Supplementary Figure S2C**). One patient had an increase in serum creatinine after diuretic dose escalation and underwent an allograft biopsy, which showed focal interstitial inflammation under the subcapsular hypo-perfused cortex and the vascular bundles in the outer medulla, and rare tubulitis. These changes were categorized as borderline changes and did not satisfy to meet the Banff criteria for acute cellular rejection. Reduction of diuretic dose and 3 days of intravenous methylprednisolone treatment led to full recovery of allograft function. After 12 months, only one patient had ACR at 17 months post-belatacept conversion. Death-censored graft survival was 100% for the entire observation period (**Supplementary Figure S2B**). No patient death occurred beyond 12 months until the end of the follow-up period (**Supplementary Figure S2C**).

A total of 13 adverse events occurred in 9 patients in the first year, which are summarized in **Supplementary Table S2**. There were 7 infection episodes, one of which required hospitalization. None of the patients had infusion reactions by 12 months.

## 4 Discussion

Our study is the first prospective study to examine the anti-proteinuric effects of CNI to belatacept conversion in KTRs with high-grade proteinuria, a group of patients that was excluded from the belatacept clinical trials (12). In this prospective interventional pilot trial, belatacept conversion was associated with a stable proteinuria and eGFR at 12 months and beyond. These changes translated into a stable predicted 7-year allograft survival despite the passage of 12 months after belatacept conversion per the abbreviated IBOX scoring, which is a validated tool for predicting allograft outcomes in KTRs (14, 15). More importantly, our 5-year extended analysis revealed a significantly stable trajectory of eGFR. Thus, our study suggests that CNI to belatacept conversion may be a potential strategy to improve long-term allograft outcomes in proteinuric KTRs. However, these findings must be validated in a larger prospective randomized study.

Although prospective studies of CNI to belatacept conversion were conducted earlier, these studies excluded patients with high-grade proteinuria. In a phase-2 randomized controlled trial, only 1 patient in both the CNI maintenance and belatacept conversion groups had proteinuria at baseline (13). In a larger randomized phase 3b trial of CNI to belatacept conversion, in which only KTRs with proteinuria <1 g/g were included, the proteinuria remained stable both in the belatacept conversion and CNI maintenance groups at a mean of 0.25 and 0.22, respectively, at 2 years; however, eGFR was maintained stable only in the belatacept conversion group and declined in CNI maintenance group (12). In another prospective study of KTRs with low-grade proteinuria, there was a higher incidence of *de novo* proteinuria in the belatacept conversion group than in the CNI maintenance group at 7 years, which was attributed to loss of hemodynamic effect of CNIs and advanced chronic changes of the allografts. However, again, a higher graft function and graft survival were observed in the belatacept conversion group (16). In our prospective study, we observed stable proteinuria in most patients and a similar preservation of graft function after belatacept conversion.

A few retrospective studies have looked at the proteinuria trend after belatacept conversion in KTRs with high-grade proteinuria. One retrospective study of belatacept conversion from CNIs or mTOR inhibitors found that conversion to belatacept was associated with decreased proteinuria at 12 months post-conversion in the subgroup of KTRs with UPCR>0.5 g/g (7). However, these findings were confounded by including KTRs that were converted from mTOR inhibitors, which are known to be associated with proteinuria (7). In another retrospective study, there was a non-significant trend towards a lower proportion of KTRs having high-grade proteinuria (UPCR >100 mg/mmol) after CNI to belatacept conversion (17). In a large retrospective study from Europe, allowing enrollment of patients with high-grade proteinuria, mean proteinuria levels were similar at baseline and 12 months post-belatacept conversion (18). However, the mean proteinuria levels were very low at baseline. Overall, the limited data in the literature suggests that CNI to belatacept conversion is not associated with significantly worsening proteinuria despite the loss of the hemodynamic and podocyte-stabilizing effects of calcineurin inhibitors, which correlates with our findings in this prospective cohort of patients.

An important question for any new anti-proteinuric therapy is whether its effect is additive to RAAS blockers and now SGLT2 inhibitors (19–21). In this study, the proteinuria remained stable in KTRs who had a similar proportion on RAAS blockers pre- and post-belatacept conversion. Since the study was conducted prior to SGLT2 inhibitors becoming approved for proteinuric kidney disease and it is still yet to be part of the standard care in proteinuric KTRs (22), none of the subjects were on SGLT2 inhibitors. Therefore, the effect of CNI to belatacept conversion on proteinuria in KTRs who are on both RAAS blockers and SGLT2 inhibitors is not known. Furthermore, while there was a tendency towards a reduction in blood pressure post-belatacept conversion, which may have also contributed to the reduction in proteinuria, the magnitude of reduction does not seem to sufficiently explain the observed findings.

The limitations of our prospective study include its small sample size and the lack of a randomized control group of CNI maintenance for comparison. Moreover, ACEI or ARBs were prescribed at the discretion of the caring physician, which might have altered the proteinuria outcomes; however, the proteinuria increased in 2 patients who newly started RAAS blockers and decreased in another patient who stopped RAAS blockers post-belatacept conversion. Another limitation is the heterogeneity of the causes of proteinuria in the groups. However, it is important to note that proteinuria is associated with adverse graft outcomes regardless of the underlying cause (2).

In conclusion, our study suggests that conversion from CNI to belatacept conversion is safe and may be associated with stable proteinuria and preserved allograft function in KTRs with high-grade proteinuria. While these findings are encouraging, the small sample size limits definitive conclusions about efficacy. This study lays the groundwork for future randomized clinical trials to further assess the safety and potential benefits of this approach on a larger scale.

## Data Availability

The raw data supporting the conclusions of this article will be made available by the authors, without undue reservation.
