# Supplementary material for: Tacrolimus to belatacept conversion in proteinuric kidney transplant recipients
Source: Front Immunol. 2024 Dec 23;15:1491514. doi: 10.3389/fimmu.2024.1491514 (PMC11701005; doi:10.3389/fimmu.2024.1491514)
Supplement: Supplementary file 1 [file Table1.docx]

**Tacrolimus to Belatacept Conversion in Proteinuric Kidney Transplant Recipients Supplementary Material**

| **Table S1.** Metabolic Parameters | Baseline + SD | 12 months + SD | P value |
| --- | --- | --- | --- |
| Systolic BP (mmHg) | 140+11 | 129+16 | 0.1272 |
| Diastolic BP (mmHg) | 76+6 | 72+9 | 0.2508 |
| Total cholesterol (mg/dL) | 177+49 | 171+49 | 0.4025 |
| LDL (mg/dL) | 96+40 | 92+41 | 0.4261 |
| HDL (mg/dL) | 52+21 | 45+20 | 0.0360* |
| Triglyceride (mg/dL) | 142+54 | 191+106 | 0.0908 |
| BMI (kg/m^2^) | 33.7+5.9 | 34.1+7.1 | 0.7795 |
| Fasting glucose (mg/dL) | 119+37 | 124+62 | 0.8089 |

**Table S1. Metabolic parameters in prospective belatacept conversion cohort.** Paired t-test is used for comparisons.

*p<0.05

| **Table S2.** Adverse events | N (%) |
| --- | --- |
| Pneumonia | 2 (13) |
| Shingles | 1 (7) |
| MRSA Cellulitis with abscess | 1 (7) |
| Cellulitis | 1 (7) |
| Pyelonephritis | 1 (7) |
| UTI | 1 (7) |
| AKI due to interstitial inflammation | 1 (7) |
| Syncope | 1 (7) |
| Basal cell carcinoma | 1 (7) |
| Recurrence of known recurrent bladder tumor | 1 (7) |
| Hypophosphatemia | 1 (7) |
| Death | 1 (7) |
| Infusion reactions | 0 (0) |
| Acute Rejection | 0 (0) |
| PTLD | 0 (0) |

**Table S2. Adverse events in prospective belatacept conversion cohort at 12 months.** Some patients had >1 event.

**Figure S1**


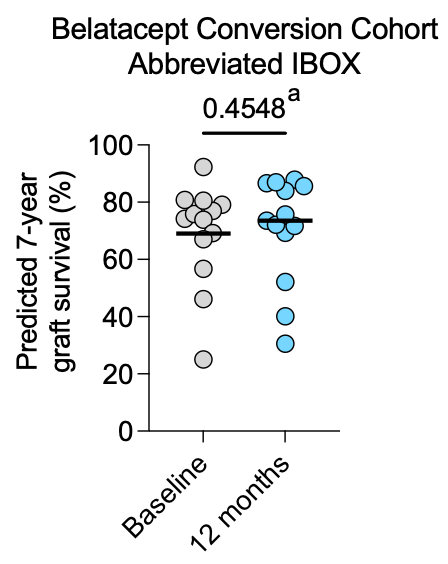


**Figure S1. Abbreviated IBOX scores for individual patients at baseline and 12 months post-belatacept conversion.**

^a^ Wilcoxon test

**Figure S2**

**Figure S2.** Patient and graph survivals in prospective belatacept conversion cohort. Kaplan Meier graphs showing A) patient, B) death-censored allograft, and C) rejection-free survivals.
